# Temporal Variation in the Essential Oil Production of *Piper aduncum* L.: Influence of Circadian Rhythms and Insights into Dillapiole Production Dynamics

**DOI:** 10.3390/plants15060976

**Published:** 2026-03-21

**Authors:** Jeferson A. S. Assunção, Camila G. Oliveira, Jessica S. Felisberto, Daniel B. Machado, Ygor Jesse Ramos, Davyson de Lima Moreira

**Affiliations:** 1 Natural Products and Biochemistry Laboratory, Rio de Janeiro Botanical Garden Research Institute, Rio de Janeiro 22460-030, RJ, Brazil; gamaacamilaa@gmail.com (C.G.O.); jessicka.salles@gmail.com (J.S.F.); danielmachadocedae@gmail.com (D.B.M.); 2 Postgraduate Program in Translational Research in Drugs and Medicines, Pharmaceutical Technology Institute (Farmanguinhos), Oswaldo Cruz Foundation (FIOCRUZ), Rio de Janeiro 21040-900, RJ, Brazil; 3 Farmácia da Terra Laboratory, Faculty of Pharmacy, Federal University of Bahia—UFBA, Salvador 40170-110, BA, Brazil; ygor.jesse@ufba.br

**Keywords:** dillapiole, medicinal plants, Piperaceae, seasonality, spatiotemporal

## Abstract

*Piper aduncum* L. (Piperaceae) is a neotropical species widely recognized for its bioactive essential oils (EOs), which exhibit antifungal, insecticidal, larvicidal, and antimicrobial properties. This study investigates the influence of circadian rhythms on the chemical composition and yield of *P. aduncum* EOs cultivated under agroecological conditions in the Rio de Janeiro Botanical Garden. Fresh leaves were collected every three hours over a 24 h cycle during both dry (July 2023) and rainy (February 2024) seasons. EOs were extracted by hydrodistillation and analyzed using GC-MS and GC-FID. A total of 20 compounds were identified in the dry season, while 10 were detected in the rainy season. Dillapiole was the predominant constituent in both periods, ranging from 75.78% to 88.27% (dry) and 75.90% to 90.86% (rainy). The highest EO yield was observed at 3:00 p.m. (0.73%) in the dry season and at 12:00 p.m. (0.61%) in the rainy season. Despite seasonal variations in chemical diversity, dillapiole content remained stable, reinforcing its biotechnological potential. The results highlight the importance of optimized harvesting times to maximize EO yield and composition, contributing to the sustainable exploitation of *P. aduncum* for medicinal and agricultural applications.

## 1. Introduction

The Piperaceae family belongs to the Magnoliidae clade, along with Aristolochiaceae and Saururaceae [1]. In Brazil, this family is represented by approximately 480 species distributed among the genera *Piper*, *Peperomia*, *Manekia*, and *Verhuellia* [2]. Among these genera, *Piper* stands out both for its high diversity and for being one of the ten largest Neotropical genera within the Magnoliidae clade, representing most species in the Piperaceae family [3]. Currently, approximately 299 species of this genus have been identified in Brazil [2].

Among the *Piper* species, *Piper aduncum* L. is particularly notable due to its widespread distribution and ability to colonize disturbed environments, playing a key role in forest regeneration [4]. Commonly known as “long pepper” and “monkey pepper”, *P. aduncum* occurs across various Brazilian biomes, including the Amazon, Caatinga, Cerrado, Atlantic Forest, Pampa, and Pantanal. Its medicinal potential is widely recognized by traditional communities and ethnobotanical studies, being used for the treatment of gynecological disorders, renal and gastrointestinal disturbances, as well as exhibiting antiseptic and wound-healing properties [5,6,7,8].

Chemically, *P. aduncum* shows a diversity of specialized metabolites, including terpenes, arylpropanoids, and flavonoids [5,7,9]. Its essential oils (EOs) are rich in dillapiole, its major constituent, followed by myristicin and apiol, and have demonstrated antibacterial, antifungal, insecticidal, and antiprotozoal activities. Several chemotypes have been described, including monoterpenes such as 1,8-cineole, β-ocimene, and γ-terpinene; the sesquiterpene *E*-nerolidol; and arylpropanoids such as safrole, sarisan and carpacin [9,10]. The EOs of *P. aduncum* are used in agriculture for pest control and in aquaculture due to its antimicrobial and insecticidal properties [10,11,12]. However, its composition may vary due to genetic, environmental, and geographical factors. Literature lacks studies addressing seasonal variation under controlled agroecological cultivation conditions [13].

The biosynthesis of bioactive compounds in plants is influenced by many biotic and abiotic factors, such as ultraviolet radiation, temperature, nutrient availability, mechanical stimuli, and seasonality [14,15]. Among these factors, the circadian rhythm has been extensively studied for its role in plant metabolic regulation. Studies suggest that the circadian cycle influences secondary metabolite production, potentially impacting the concentration of bioactive substances throughout the 24 h [16,17].

Despite the significance of the circadian rhythm in metabolite production in medicinal plants, few studies have explored its influence on the chemical composition and yield of *P. aduncum* EOs. An exception is the work by Morandim et al. [18], which investigated the circadian variation in the biosynthesis of antifungal prenylated chromenes in *P. aduncum* leaves, showing that the enzymatic activity of prenyltransferase is regulated by time of day. Given this gap, the present study aims to evaluate the impact of the circadian rhythm on the production and chemical composition of *P. aduncum* EOs cultivated under agroecological conditions. To achieve this, leaves were collected at different time points over a 24 h cycle during both dry and rainy seasons, with the goal of identifying temporal variation patterns in the biosynthesis of its constituents. The findings may provide insights to optimize the agroecological management and harvesting of *P. aduncum*, maximizing its productive efficiency and biotechnological potential.

## 2. Results

### 2.1. Climatic Variables and Essential Oil Yield

**Figure 1:**
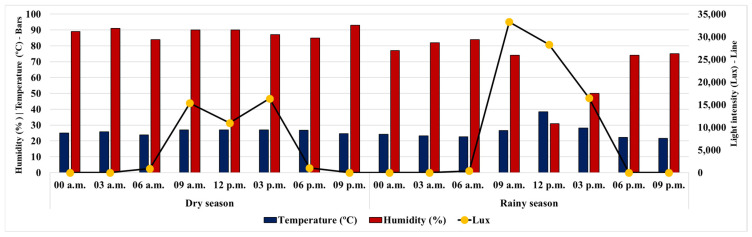
Climatological averages by leaf harvesting time for *Piper aduncum* over a 24 h period in July 2023 and February 2024.

**Figure 2:**
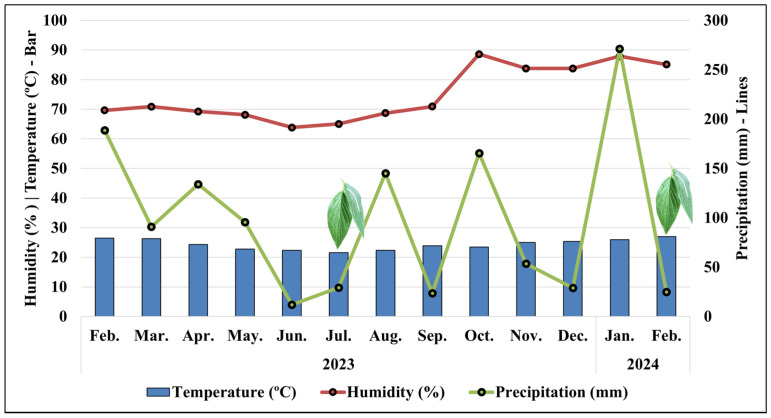
Monthly climatological averages over one year for the study of the circadian cycle of *Piper aduncum*, with reference to July 2023 and February 2024. Leaf symbols indicate the study months (July—dry season; February—rainy season).

The monthly and 24 h climate averages, recorded simultaneously with the harvesting of *P. aduncum* leaves every 3 h, reflect the typical environmental conditions of the evaluated periods, as shown in Figure 1 and Figure 2. These data confirm that the *P. aduncum* specimen was indeed subjected to the characteristic conditions of the dry and rainy seasons during the circadian cycle study.

Figure 1 shows that during the dry season, relative humidity (RH) remained relatively stable over the 24 h collection period, ranging from 85% to 93%. The lowest RH was recorded at 6 a.m. (85%) and the highest at 9 p.m. (93%). The lowest temperature (23.8 °C) also occurred at 6 a.m., coinciding with the lowest RH. In contrast, the highest temperature (27 °C) was observed at 9 a.m., when RH was 90%. Although the peaks of temperature and RH did not coincide, the RH variation throughout the day was only 8%, indicating low hydric amplitude during this period. The lowest light intensity was recorded at 6 a.m. (895 lux), accompanying the lowest temperature and RH, while the highest value occurred at 3 p.m. (16,340 lux).

During the rainy season, unlike the dry season, RH showed high variation, ranging from 31% to 84%. The lowest value was recorded at 12 p.m. (31%) and the highest at 6 a.m. (84%). The lowest temperature (21.7 °C) was observed at 9 p.m., while the highest (38.3 °C) occurred at 12 p.m., coinciding with the lowest RH. Similarly, the lowest light intensity was recorded at 6 a.m. (400 lux), and the highest at 9 a.m. (33,300 lux).

Figure 2 shows that the dry season was well established prior to leaf harvesting. The months of May and June 2023, which preceded the leaf harvest in July, recorded low rainfall levels, with 95.6 mm in May and only 12 mm in June. During the harvest month (July), precipitation remained low, totaling 29.2 mm. The average monthly temperature and RH during this period were 21.6 °C and 65%, respectively.

In contrast, during the rainy season, the leaf harvest was preceded by a period of high rainfall, with a total of 271 mm in January 2024, followed by 24.8 mm in February. The average monthly temperature and RH reached 27 °C and 85.1%, respectively.

**Figure 3:**
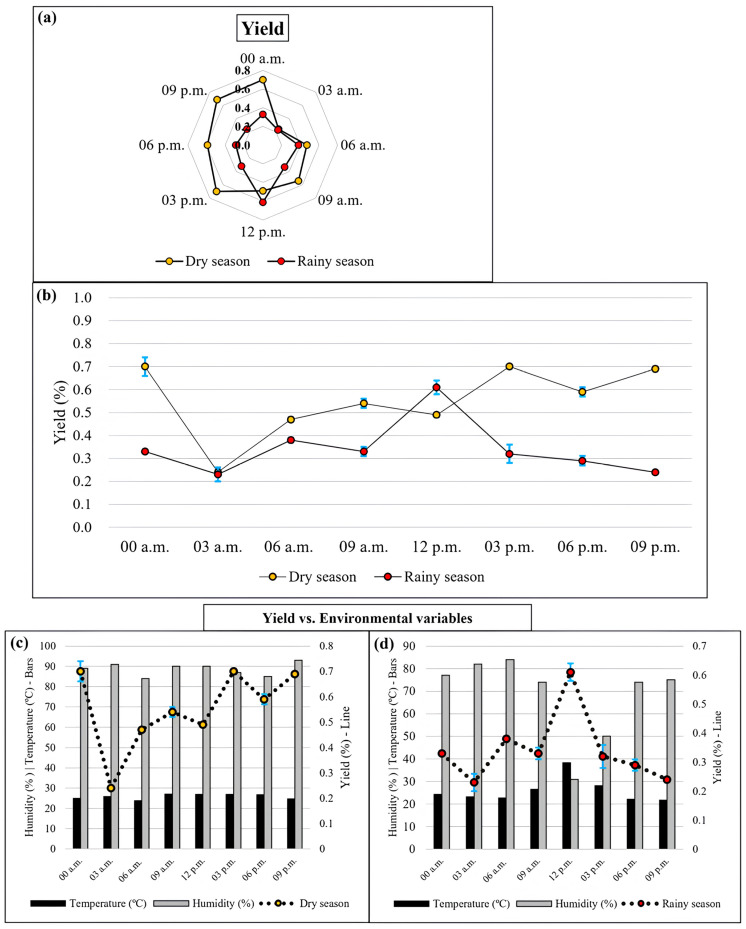
Essential oil yields (%) obtained from samples collected every three hours, comparing daily variations and distribution in the circadian cycle of Piper aduncum with climatological normals for humidity (%) and average temperature (°C) in July 2023 and February 2024. Legend: (**a**,**b**) changes in the essential oil yields from the leaves of Piper aduncum in both periods; (**c**,**d**) climatological normals compared to essential oil yield over 24 h in July 2023 and February 2024; blue vertical error bar: Indicates the standard deviation of the analyzed samples.

A total of 16 EOs samples were obtained, with eight collected during the dry season and eight during the rainy season. Results are shown in Figure 3.

The radar chart (Figure 3a), which illustrates the distribution of yields over 24 h, highlights a more uniform distribution in July (dry) compared to February (rainy).

Considering the dry season, the highest values were recorded between 12 a.m. (0.71 ± 0.04%) and 3:00 p.m. (0.73 ± 0.01%), while the lowest yield was registered at 3:00 a.m. (0.24 ± 0.01%). Statistical analysis revealed significant differences (*p* < 0.05) between harvesting times, except for the comparisons between 12 a.m. and 3:00 p.m.

During the rainy season, the lowest yield was registered at 3 a.m. (0.23 ± 0.03) and 9:00 p.m. (0.24 ± 0.01%), while the highest was recorded at noon (0.61 ± 0.03%). As in the dry season, statistically significant differences (*p* < 0.05) were found between harvesting times, except for 12:00 a.m., 9:00 a.m., and 3:00 p.m., as well as between 3:00 a.m. and 9:00 p.m. When comparing the yields from both periods, a statistically significant difference (*p* < 0.05) was consistently registered.

### 2.2. Variation of the Volatile Compounds over the Circadian Cycle

The chemical composition of *P. aduncum* EOs was analyzed over a 24 h cycle during the dry and rainy seasons. The detailed chemical composition is presented in Table 1 and Table 2. In Supplementary Material Figure S1, the mass spectrum of the major compound present at all harvest times in both analyzed periods, dillapiole, is provided.

**Figure 4:**
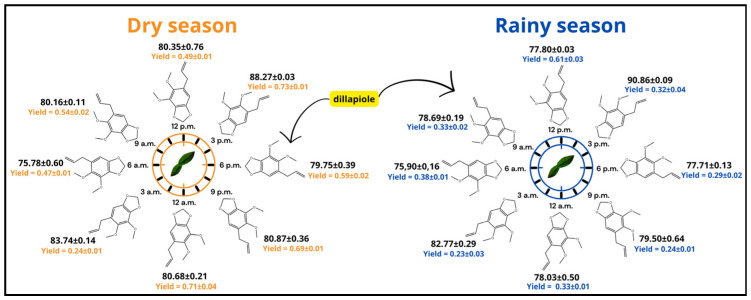
Variation of the arylpropanoid dillapiole and essential oil yield from *Piper aduncum* leaves throughout the circadian cycle, assessed during the dry season (July 2023) and the rainy season (February 2024). Results are shown as average ± Standard deviation for both percentage and yield.

In the dry season, up to 20 substances were identified throughout the circadian cycle, with variations ranging from 5 to 20 components at different analyzed times. The total content of identified substances changed from 91.84% to 96.59%, indicating a comprehensive chemical profile. During this period, the plant was in its reproductive phase, as mentioned in the Experimental section, and showed a chemical composition predominantly composed of arylpropanoids, particularly dillapiole, whose content ranged from **75.78 ± 0.60**% to **88.27 ± 0.03**% (Figure 4) (Table 1).

In relation to the rainy season, 10 substances were identified, with variations ranging from 5 to 9 at different analyzed times. The total content of identified substances changed from 88.64% to 99.98%, also considered adequate. As in the rainy season, the plant remained in the reproductive phase, displaying a chemical profile dominated by dillapiole, with content ranging from **75.90 ± 0.16**% to **90.86 ± 0.09**% (Figure 4) (Table 2).

The arylpropanoid dillapiole remained the major compound in the EOs of *P. aduncum* in both analyzed seasons, with content consistently above **75%**. However, chemical diversity was higher during the dry season, with up to 20 substances identified throughout the circadian cycle, whereas in the rainy season, this number was reduced to 10. It is worth noting that during the dry season, exactly at 3:00 p.m., when dillapiole reached its highest content in the EO, only five distinct compounds were identified. The same pattern was observed during the rainy season, where at the same time, only four different compounds were registered. This result suggests a possible dominance of the shikimate pathway.

Among the substances with content equal to or greater than 1%, the non-oxygenated monoterpene β-pinene showed considerable variations throughout the day. Its highest content was registered at 6:00 a.m. (1.85 ± 0.09%), while it was absent at 6:00 p.m., with the lowest value recorded at 3 a.m. (1.05 ± 0.02%). The monoterpene *Z*-β-ocimene showed its peak content in the early morning hours, reaching 1.86±0.35% at 6:00 a.m., before a sharp decline throughout the day and becoming completely absent at 3:00 p.m. Its lowest percentage was recorded at 3:00 a.m. (0.81 ± 0.12%). Similarly, *E*-β-ocimene showed the highest content during the daytime, with values of 4.67 ± 1.19% at 6:00 a.m. and 4.37 ± 0.06% at 12:00 p.m., followed by complete absence at 6:00 p.m. In the rainy season, *Z*-β-ocimene peaked later, at 6:00 p.m. (2.34 ± 0.04%).

Among the sesquiterpenes, *E*-caryophyllene reached its highest content at 9:00 a.m. (2.83 ± 0.01%) in the dry season, but remained relatively high throughout the night, showing statistically significant differences (*p* < 0.05) compared to other time points. On the other hand, bicyclogermacrene exhibited an opposite pattern to the other substances in the dry season, with its highest relative percentage recorded in the early hours of the morning (1.90 ± 0.01% at 12:00 a.m.), followed by a decrease at 3:00 a.m. (1.24 ± 0.13%). The measurements taken at these two times were significantly different (*p* < 0.05) from each other and from those taken at the other periods of the day, which did not show relevant statistical differences. It is important to note that bicyclogermacrene reached its highest peak of production at 12:00 a.m. during the dry season, while in the rainy season, the highest content was observed at dawn and nighttime (6:00 a.m., 9:00 a.m. and 9:00 p.m.). This suggests that bicyclogermacrene follows a circadian biosynthesis pattern, being preferentially accumulated during periods of lower solar radiation in the rainy season.

**Figure 5:**
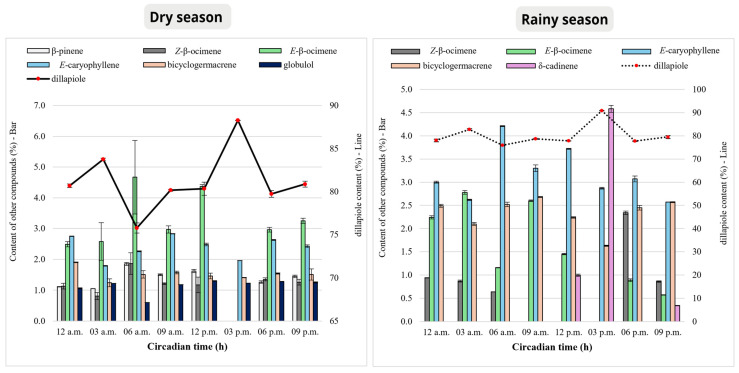
Percentage variation of compounds with relative percentage above 1% in the essential oil of *Piper aduncum* leaves throughout the circadian cycle in the dry (July 2023) and rainy (February 2024) seasons.

The non-oxygenated sesquiterpene δ-cadinene displayed a highly variable pattern, being absent at several time points (12:00 a.m., 3:00 a.m., 9:00 a.m., and 6:00 p.m.) and showing the highest percentage among non-major substances at 3:00 p.m. (4.58 ± 0.07%), coinciding with the peak content of dillapiole (Figure 5).

The oxygenated sesquiterpene globulol maintained relatively stable content throughout the circadian cycle during the dry season (1.06 ± 0.02% to 1.30 ± 0.01%), with its highest production peak recorded at 12:00 p.m. (1.30 ± 0.01%). Although the variations were not highly pronounced, statistical analyses indicated significant differences (*p* < 0.05) among the evaluated time points. Notably, globulol was not identified in any samples during the rainy season, which may suggest a drought response, with its production being suppressed under higher humidity conditions (Figure 5).

### 2.3. Correlations and Statistical Analyses

Based on Pearson’s correlation presented in Table 3, significant relationships with varying percentages were identified among the variables analyzed throughout the circadian cycle. These relationships involved interactions among substances and environmental factors affecting yield.

During the dry season, Pearson’s correlations revealed significant interactions among the substances (*p* < 0.05). β-pinene showed a very strong positive correlation with *Z*-β-ocimene (*r*^2^ = 0.942) and *E*-β-ocimene (*r*^2^ = 0.973), while these latter two substances also exhibited a very strong positive relationship with each other (*r*^2^ = 0.908). Other substances, such as *E*-caryophyllene and bicyclogermacrene, displayed a strong positive correlation (*r*^2^ = 0.781). This is expected because these compounds are generated in the same biosynthetic pathway.

On the other hand, dillapiole showed very strong negative correlations with three substances: β-pinene (*r*^2^ = −0.932), *Z*-β-ocimene (*r*^2^ = −0.992), and *E*-β-ocimene (*r*^2^ = −0.904). This may suggest a dichotomy in the production of monoterpenes and arylpropanoids. In other words, when the shikimate pathway is active in *P. aduncum*, the acetate–mevalonate pathway is suppressed.

The oxygenated sesquiterpenes globulol and ledol also demonstrated a very strong negative correlation with each other (*r*^2^ = −0.925). This variation pattern is expected, since the two compounds are isomers.

Moderate correlations were observed among the analyzed substances. β-pinene exhibited positive correlations with *E*-caryophyllene (*r*^2^ = 0.469) and ledol (*r*^2^ = 0.452). *Z*-β-ocimene also showed positive correlations with *E*-caryophyllene (*r*^2^ = 0.495) and ledol (*r*^2^ = 0.610) but exhibited a negative correlation with globulol (*r*^2^ = −0.545). In turn, *E*-β-ocimene had a positive correlation with ledol (*r*^2^ = 0.508) and a negative correlation with globulol (*r*^2^ = −0.404). Dillapiole stood out by showing negative correlations with *E*-caryophyllene (*r*^2^ = −0.533) and ledol (*r*^2^ = −0.638), while maintaining a positive correlation with globulol (*r*^2^ = 0.586). However, these correlations may have neither biosynthetic nor ecological significance, as they are weak.

When analyzing the correlation matrix between substances and environmental variables, no very strong relationships were identified, only correlations ranging from very weak to moderate. The only strong correlation observed was between globulol and temperature (*r*^2^ = 0.732).

Among the moderate correlations, the most notable were: β-pinene, which showed a negative correlation with temperature (*r*^2^ = −0.402); the non-oxygenated monoterpene limonene, which had a negative correlation with humidity (*r*^2^ = −0.477); and *Z*-β-ocimene, which exhibited similar negative correlations with temperature (*r*^2^ = −0.535) and light intensity (lux) (*r*^2^ = −0.532). Additionally, *E*-β-ocimene displayed a negative correlation with temperature (*r*^2^ = −0.408), while globulol showed a positive correlation with humidity (*r*^2^ = 0.558). Ledol demonstrated negative correlations with both temperature (*r*^2^ = −0.630) and humidity (*r*^2^ = −0.678). Conversely, dillapiole exhibited positive correlations with temperature (*r*^2^ = 0.504) and light intensity (*r*^2^ = 0.455).

Analyses between chemical classes and environmental variables did not reveal significant relationships, as was also the case for individual compounds and these variables. The most notable correlation was observed between arylpropanoids and temperature (*r*^2^ = 0.519), as well as light intensity (*r*^2^ = 0.448). This result was expected, given that dillapiole, the main compound in *P. aduncum* EO, exhibited a similar correlation profile when analyzed against the same environmental variables.

**Figure 6:**
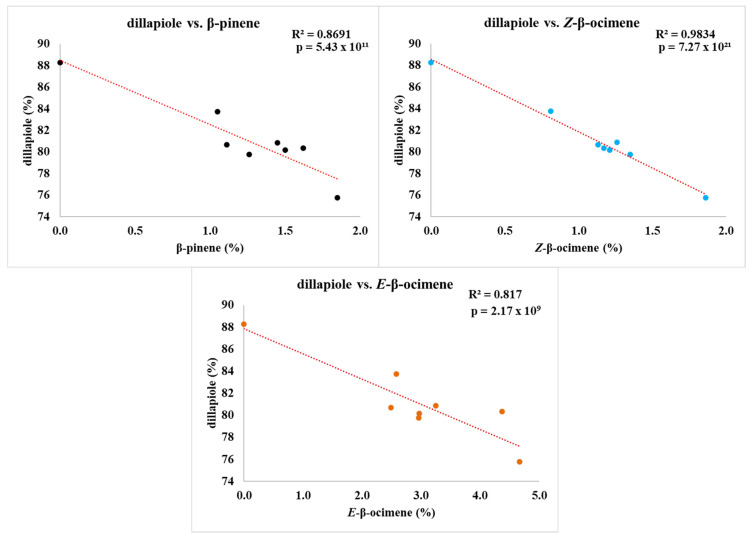
Correlation between dillapiole content and the compounds β-pinene, *Z*-β-ocimene, and *E*-β-ocimene in the essential oils of *Piper aduncum* during the dry season (July 2023).

The scatter plots (Figure 6) illustrate that more than 80% of the variation in dillapiole content can be explained by its inverse relationship with three other substances: *Z*-β-ocimene, which showed the strongest antagonism, followed by β-pinene and *E*-β-ocimene, as mentioned before. We hypothesize a dichotomy between the shikimate and acetate–mevalonate pathways.

As observed in the dry season, the rainy season also revealed significant interactions among the analyzed compounds in *P. aduncum* EOs, with strong and very strong correlations (Table 4). One notable relationship was the strong positive correlation between δ-cadinene and dillapiole (*r*^2^ = 0.877), indicating that an increase in one is directly associated with an increase in the other. Conversely, both δ-cadinene and dillapiole exhibited strong negative correlations with bicyclogermacrene. The correlation between δ-cadinene and bicyclogermacrene was *r*^2^ = −0.858, while that between dillapiole and bicyclogermacrene reached *r*^2^ = −0.885. These results support the hypothesis that the increased levels of δ-cadinene and dillapiole are proportionally associated with a decrease in bicyclogermacrene content. However, it is difficult to explain, as it may be related to the activation of enzymes in biosynthetic pathways, chemical defense, volatilization driven by the vapor pressure of these compounds, among many other variables.

Among the moderate correlations, δ-cadinene showed negative correlations with *Z*-β-ocimene (*r*^2^ = −0.447) and *E*-β-ocimene (*r*^2^ = −0.630), while dillapiole displayed a negative correlation with *E*-caryophyllene (*r*^2^ = −0.514).

When analyzing the correlation matrix between compounds, environmental variables, and EO yield, it was observed that, during the rainy season, fewer compounds with content above 1% exhibited strong or very strong correlations compared to the dry season. Notably, *E*-caryophyllene showed a strong positive correlation with light intensity (*r*^2^ = 0.883). EO yield also exhibited significant correlations, including a strong positive correlation with temperature (*r*^2^ = 0.892) and a strong negative correlation with relative humidity (*r*^2^ = −0.764).

Among the moderate correlations, *Z*-β-ocimene stood out, showing associations with all analyzed environmental variables: temperature (*r*^2^ = −0.603), humidity (*r*^2^ = 0.488), and light intensity (*r*^2^ = −0.523). Similarly, δ-cadinene displayed a negative correlation with humidity (*r*^2^ = −0.585), while dillapiole exhibited a negative correlation with light intensity (*r*^2^ = −0.477). On the other hand, bicyclogermacrene showed a positive correlation with humidity (*r*^2^ = 0.498).

During the rainy season, as in the dry season, no significantly strong correlations were observed between chemical classes and environmental variables. However, unlike the dry season, the strongest correlations were recorded for non-oxygenated monoterpenes, which exhibited moderate negative correlations with temperature (*r*^2^ = −0.531) and light intensity (*r*^2^ = −0.529), along with a strong positive correlation with humidity (*r*^2^ = 0.725). In contrast, non-oxygenated sesquiterpenes showed the opposite trend, with moderate positive correlations with temperature (*r*^2^ = 0.501) and light intensity (r^2^ = 0.403), but a stronger negative correlation with humidity (*r*^2^ = −0.620).

These findings highlight the significant influence of certain environmental variables during the rainy season, although this influence is limited to specific compounds.

**Figure 7:**
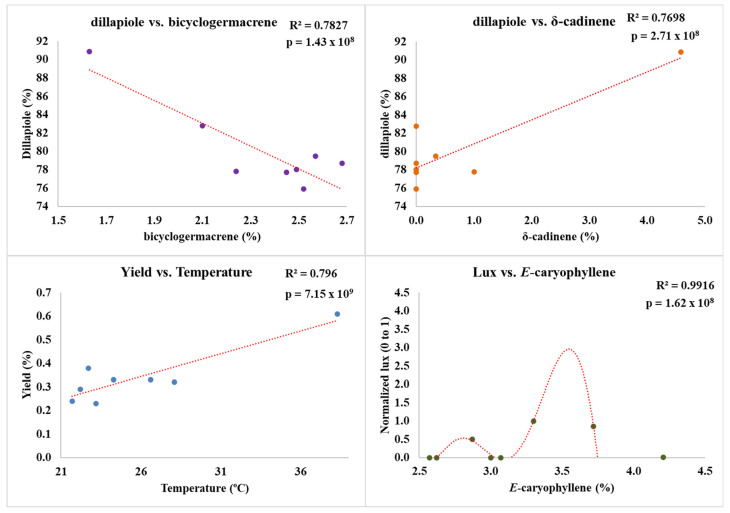
Scatter plots of strongest correlation: dillapiole content and the compounds bicyclogermacrene and δ-cadinene; yield vs. temperature; lux vs. *E*-caryophyllene. Rainy season (February 2024). Legend: Colored points represent individual experimental samples.

The scatter plots (Figure 7) visually reinforce these correlations, showing trend lines that explain more than 75% of the observed variation in compound content and EO yield in relation to environmental variables. However, in the case of *E*-caryophyllene, despite its strong correlation with light intensity, the pattern observed in the graph suggests a non-linear relationship. This behavior indicates the possibility that additional factors, beyond light incidence, may be influencing *E*-caryophyllene content, which were not addressed within the scope of this study.

The correlation matrix presented in Table 5 exhibits significant results among the substances from the dry and rainy periods, revealing distinct patterns. These results indicate seasonal patterns, where some substances appear to be synthesized together, while others behave inversely.

Strong positive correlations were observed between different compounds in both the dry and rainy seasons, suggesting a possible shared biosynthetic influence. Notably, limonene was detected only during the dry season at a single time point (6:00 p.m., 1.03 ± 0.22%) and was entirely absent in all samples from the rainy season. This suggests that limonene may be an environmentally sensitive compound, not being produced under high humidity or lower solar radiation. Additionally, limonene from the dry season showed a strong correlation with *Z*-β-ocimene from the rainy season (*r*^2^ = 0.845), while ledol from the dry season was highly correlated with *E*-caryophyllene from the rainy season (*r*^2^ = 0.749). Additionally, β-pinene from the dry period exhibited a strong association with bicyclogermacrene from the rainy period (*r*^2^ = 0.835), while *Z*-β-ocimene and *E*-caryophyllene from the dry period also showed high correlations with bicyclogermacrene from the rainy period (*r*^2^ = 0.860 and *r*^2^ = 0.746, respectively). The relationship between dillapiole from the dry period and δ-cadinene from the rainy period (*r*^2^ = 0.787) further supports the hypothesis of a chemical defense between these two compounds, but the evidence is too weak to support this. More investigation is needed.

One of the most remarkable correlations was the very strong positive relationship between dillapiole in both periods (*r*^2^ = 0.953), indicating stability in its presence regardless of seasonality. However, dillapiole from the rainy period was the only substance to exhibit very strong negative correlations, particularly with β-pinene (*r*^2^ = −0.937) and *Z*-β-ocimene (*r*^2^ = −0.954), in addition to a strong negative correlation with *E*-β-ocimene (*r*^2^ = −0.899).

Among the most relevant negative correlations, the strong inverse relationship between bicyclogermacrene from the rainy period and dillapiole from the dry period (*r*^2^ = −0.843) stands out, as well as between *E*-caryophyllene from the dry period and dillapiole from the rainy period (*r*^2^ = −0.640). Other important negative correlations include *E*-β-ocimene from the dry period with δ-cadinene from the rainy period (*r*^2^ = −0.744), *Z*-β-ocimene from the dry period with δ-cadinene from the rainy period (*r*^2^ = −0.829), and β-pinene from the dry period with δ-cadinene from the rainy period (*r*^2^ = −0.825). Furthermore, *E*-caryophyllene from the rainy period demonstrated a significant inverse relationship with globulol from the dry period (*r*^2^ = −0.671), while dillapiole from the dry period exhibited a moderate negative correlation with *E*-caryophyllene from the rainy period (*r*^2^ = −0.664). The analyses indicate a possible antagonism in the biosynthesis of these substances, possibly influenced by seasonal variations.

It is worth noting that δ-cadinene was absent at almost all time points during the dry season, except at 12:00 p.m. (1.00 ± 0.02%), 3:00 p.m. (4.58 ± 0.07%), and 9:00 p.m. (0.34 ± 0.01%), and was completely absent in the rainy season. This pattern suggests that its production may be highly specific, influenced by environmental stimuli such as humidity or variations in light intensity. Similarly, γ-cadinene followed the same trend, being present during the dry season in six out of eight time points but nearly disappearing in the rainy season, where it was detected only at 6:00 a.m. (0.54 ± 0.00%) and 9:00 p.m. (0.33 ± 0.03%), both periods of lower light incidence, with very low content. This behavior may be linked to water stress, with its production being suppressed under higher humidity conditions.

**Figure 8:**
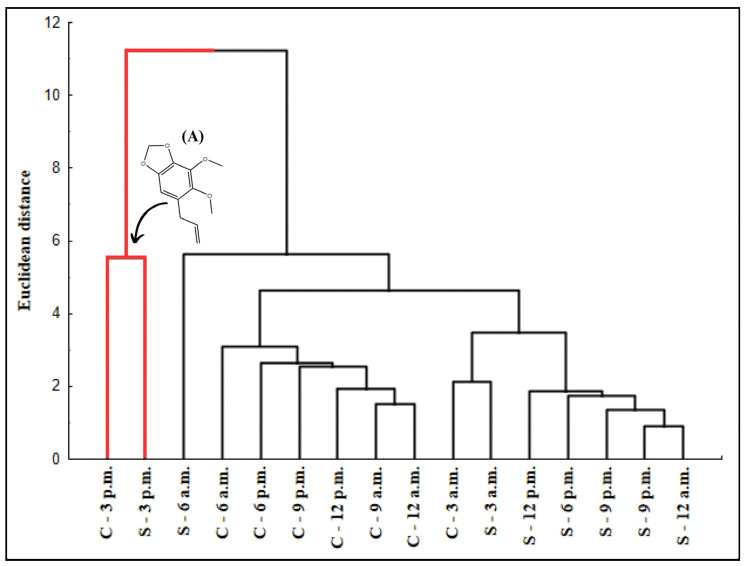
Hierarchical Cluster Analysis (HCA) representing the similarity between the compounds in the essential oils of *Piper aduncum* leaves during the circadian cycle in the dry (S) and rainy (C) seasons over a 24 h period. (A) Dillapiole (red color).

**Figure 9:**
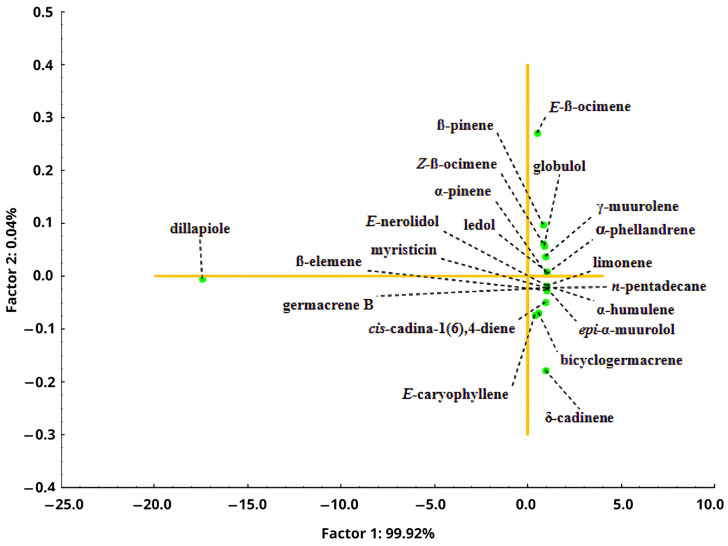
Principal Component Analysis (PCA) of the sampling times of essential oils from *Piper aduncum* leaves throughout the circadian cycle, in the dry and rainy seasons over a 24 h period. Legend: Yellow lines: indicate the zero axes (origin) of the principal components, dividing the factor plane into quadrants; Green dots: represent individual variables (chemical compounds).

The chemical composition assessment of *P. aduncum* leaf EOs during the dry and rainy periods was conducted using Hierarchical Cluster Analysis (HCA), represented in Figure 8, and Principal Component Analysis (PCA), illustrated in Figure 9. For statistical analysis, only substances present at concentrations above 0.05% were considered.

The analyzed dendrogram (Figure 8) reveals the formation of two distinct groups. Generally, the Euclidean distance indicates low variation (0 to 12) among most samples. However, a more significant separation was observed between two groups, with a distance of 11.22. The first group contains only dry 3:00 p.m. and rainy 3:00 p.m., which showed *a higher percentage of dillapiole and lower chemical diversity*, whereas the second group displayed *greater substance diversity and lower dillapiole content*.

Despite the separation in HCA, PCA (Supplementary Materials Figure S2) shows that harvest times grouped exclusively on the left side, regardless of the analyzed period. However, the slight dispersion observed suggests that some substances may exhibit distinct variation patterns.

Dillapiole (−17.43 eigenvalues) appears isolated from the other substances, being primarily explained by Factor 1 = 99.92%, confirming its influence by different factors compared to the other compounds, which are mostly aligned with Factor 2 = 0.04% (Figure 9). The substances *E*-β-ocimene and δ-cadinene, located at the extremes of the graph, exhibited factor loadings of 0.27 and −0.17 eigenvalues, respectively, indicating a low influence on the overall variability of the other substances.

The results indicate that although the chemical composition of *P. aduncum* EOs is influenced by interactions between metabolites and environmental variables throughout the circadian cycle, dillapiole remains at high amounts, as does the EOs yield, particularly during the dry season. This stability, combined with a high yield, enhances the feasibility of large-scale production, expanding its applications across various sectors and adding value to a species native to the Atlantic Forest. The predominance of dillapiole in all samples, regardless of seasonal and hourly variations, underscores its metabolic and ecological significance. Fluctuations in its content during the rainy season suggest an adaptive response of the plant to environmental changes, highlighting the influence of factors such as solar radiation and temperature on the biosynthesis of secondary metabolites. It is important to highlight that this study was conducted using a single cultivated specimen of *P. aduncum*, which paves the way for future large-scale studies involving multiple individuals to better understand the species’ population dynamics under cultivation conditions.

Although the present chronochemical assessment was conducted on a single cultivated specimen, this individual derived from a wild matrix propagated by stem cuttings; additional plants are currently under vegetative propagation (~4 individuals) to enable replicated temporal campaigns and to test the generality of the observed patterns across individuals and genotypes.

## 3. Discussion

### 3.1. Climatic Variables During Leaf Sampling

Our hypothesis is that the observed humidity pattern reflects the microclimate buffering effect typical of tropical forests. In this case, the structural configuration of the trees and local temperature dynamics regulate relative humidity independently of rainfall seasonality [19,20,21].

### 3.2. Variation in Volatile Compounds and Essential Oil Yield

Overall, the results indicate that *P. aduncum* showed variations in EOs yield according to the circadian cycle, allowing the identification of optimal harvesting times at **3:00 p.m. and 12 a.m. during the dry season and 12 p.m. in the rainy seasons**. These patterns highlight the influence of daily metabolic rhythms on essential oil (EO) production. The high yield registered in the dry season is in accordance with studies on other species of the *Piper* genus, such as *Piper cernuum* Vell. [22], *P. gaudichaudianum* Kunth [23], *P. mollicomum* Kunth [24], and *P. divaricatum* G. Mey. [25].

Nevertheless, the findings of this study contrast with previous reports, which observed a higher EO yield in *P. amalago* L. during the rainy season (0.07%) and a decrease in the dry season (0.06%) [26]. This result may be related to the plant’s phenological stage, as the analyzed specimen was in the flowering-to-fruiting phase and located in a fragment of the Atlantic Forest in Espírito Santo [27]. In contrast, *P. aduncum* in the present study showed continuous flowering and fruiting once it reached maturity, suggesting that EO production may occur independently of strict seasonal phenological changes for this species.

Within this metabolic framework, the constant presence of dillapiole suggests that this compound plays a fundamental role in the plant’s ecology, possibly contributing to its defense against herbivores, pathogens, or plant competitors. Previous studies highlight its antifungal and insecticidal properties, reinforcing its importance as an adaptive response trait [28,29,30]. Moreover, the maintenance of dillapiole production under different environmental conditions suggests that its biosynthetic pathway is highly prioritized, ensuring a continuous supply of this essential metabolite for the species’ survival. Despite its consistent presence, variation in dillapiole content was observed. This suggests that environmental factors, such as temperature and solar radiation, directly influence dillapiole biosynthesis, reinforcing studies on the impact of abiotic conditions on the production of secondary metabolites in medicinal plants. Among these factors, light radiation plays a crucial role in regulating plant metabolism, directly affecting the biosynthesis of chemical compounds [31,32,33]. In this study, for example, the lower dillapiole content at 6 a.m. and 6:00 p.m. (dry and rainy seasons) may be associated with light intensity. Besides the reduction in the dillapiole content at 12 p.m., under the peak of solar light, *P. aduncum* may redirect its metabolic resources toward the production of other protective compounds, such as flavonoids and carotenoids, which assist in mitigating oxidative stress [31,32]. Studies show that prolonged light irradiation can significantly increase the production of flavonoids and phenolic acids, substances directly linked to protection against oxidative stress [34,35].

Beyond seasonality and light radiation, the chemical composition of plant species can also be affected by other factors. These include chemical stress, caused by the accumulation of salt, gaseous toxins, pollutants, heavy metals, and pesticides; mechanical stresses, such as wind action and soil movement; and biotic factors resulting from interactions with nematodes, bacteria, viruses, and fungi [36,37].

From an applied perspective, the peak content of dillapiole at **3:00 p.m.** highlights this time as the most suitable for optimized extraction of the compound. Dillapiole shows biologically significant properties, including antifungal and insecticidal activity. Studies indicate that it inhibits the symbiotic fungus of leaf-cutting ants, suggesting its potential use as a companion plant in crops susceptible to this type of pest [29]. Furthermore, its antifungal activity against *Botryodiplodia theobromae* and *Colletotrichum acutatum* reinforces its potential as an alternative to synthetic pesticides, with possible applications in biocontrol strategies [30].

Although the high relative percentage of dillapiole in the EOs of *P. aduncum* has been widely reported across different regions of Brazil, such as Pará (73%—[38]), Amazonas (80%—[39]; 80%—[40]), Acre (78%—[28]), and São Paulo (81%—[41]), its consistent presence in the species, regardless of environmental and geographical variations, highlights its ecological significance and biotechnological potential. However, further studies are needed to fully assess the stability of the chemical profile under different environmental conditions and determine whether it remains unchanged despite plasticity events.

In contrast to dillapiole, compounds such as β-pinene, *Z*-β-ocimene, and *E*-β-ocimene were strongly influenced by the circadian cycle, showing higher content in the morning and a sharp decline at 3:00 p.m., when dillapiole reached its peak. This pattern suggests a dynamic allocation of metabolic resources throughout the day. In addition to daily variation, these metabolites may also be influenced by different cultivation techniques, as variations in their content have been observed in *Piper divaricatum* G. Meyer cultivated using different propagation methods, including stem cuttings and tissue culture, analyzed over 30, 60, and 90 days [42].

### 3.3. Correlations Among Essential Oil Constituents, Yield, and Abiotic Factors

The very strong correlations between dillapiole and the monoterpenes β-pinene, *Z*-β-ocimene, and *E*-β-ocimene, as well as the moderate correlations among oxygenated sesquiterpenes, may be explained by the internal metabolic control of *P. aduncum*. This control may prioritize the biosynthesis of one compound over another. Sesquiterpenes are biosynthesized through the mevalonic acid pathway (MVA), while hemiterpenes, monoterpenes, and diterpenes can be biosynthesized via either the MVA or the methylerythritol phosphate pathway (MEP) in plant cells [43]. In contrast to the arylpropanoid dillapiole, it is biosynthesized via the shikimic acid pathway (SAP), responsible for producing some phenolic substances, including lignoids and flavonoids [44]. Accordingly, different biosynthetic pathways may compete for precursor molecules as well as cofactors, which implies a possible regulatory effect capable of altering the biosynthesis of certain compounds [45]. In general, volatile compounds present in plant species can be altered by internal signals, affecting the availability of substances in plants [43]. Furthermore, it is evident that the proportion of non-oxygenated sesquiterpenes decreased more sharply during the rainy season. In the dry season, the total content of non-oxygenated sesquiterpenes ranged from 4.28% to 6.23%, whereas in the rainy season, it increased to values between 5.01% and 9.12%. These findings suggest that sesquiterpenes are not as stable as dillapiole and may be more strongly influenced by environmental conditions. These findings reinforce the hypothesis that the plant regulates its biosynthesis to favor certain compounds over others, even when their metabolic pathways differ [43,45].

Regarding cultivated *Piper* species, *Piper umbellatum* L. and *P. gaudichaudianum* Kunth showed distinct responses to environmental conditions. While *P. umbellatum* revealed higher EO yield when cultivated in shade [46], *P. gaudichaudianum* demonstrated a strong correlation between its compound composition and environmental factors, including relative humidity, temperature, and light intensity [47].

In this context, based on the metabolic profile analyses conducted during the dry and rainy periods, it was possible to confirm that the *P. aduncum* specimen analyzed belongs to the dillapiole chemotype. This is the first recorded identification and study of the dillapiole chemotype of *P. aduncum* in the territory of Rio de Janeiro State. To date, no studies were found in scientific literature analyzing the EOs of *P. aduncum* under the conditions described herein, making the present work a pioneer in the State of Rio de Janeiro. The research group has already developed three relevant studies with this specimen: one focused on optimizing dillapiole production by ultraviolet-C radiation exposure during post-harvest leaf processing [11]; another that confirmed its insecticidal activity against *Ctenocephalides felis felis* [48]; and a third that provided the first chemical fingerprint of an antimycobacterial sample of *P. aduncum* [6]. Consistent with this novelty, literature reviews indicate a lack of previous studies on this chemotype in the region. However, it has been observed that the dillapiole chemotype is widely distributed in areas closer to the equator [10,49,50]. A literature review on the major chemical classes and substances studied worldwide highlighted that arylpropanoids, particularly dillapiole, primarily biosynthesized by *P. aduncum*, are found in higher contents in regions near the equator [49].

The correlation between the dillapiole and local environmental variables did not show significant relationships, supporting the stability of the EO for the production of this arylpropanoid. This allows for cultivation with minimal effort. Although dillapiole exhibited very strong correlations with other substances, its relatively high percentage remained, ranging between 75% and 90% throughout the circadian cycle in both analyzed periods (dry and rainy). These values are consistent with those found in various tropical regions, such as the Asian Tropics (Malaysia), Central America (Cuba), and South America (Amazon Rainforest and Atlantic Forest) [10,49].

Although EO yields are moderately to strongly influenced by environmental variables during the rainy season, the recorded yield remains adequate for production. The optimization of EO yield from 50 g of *Piper nigrum* L. seeds reached 0.75% [51]. Seasonal and circadian analyses of *P. gaudichaudianum* Kunth leaves reported yields ranging from 0.02% to 0.12% and from 0.10% to 0.23%, respectively, using 100 g of leaves per sample [47]. Similarly, EO yields of *P. rivinoides* Kunth varied between 0.16% and 0.63% across different seasons, also based on 100 g of leaves [52]. In addition, the standard EO yield of *Matricaria recutita* L. reached 0.88% from 189 g of flowers [53]. In comparison, the highest EO yield of *P. aduncum*, extracted from only 50 g of fresh leaves, was remarkably close to or higher than these values, reaching 0.73%, highlighting the leaves as the most abundant plant organ.

Finally, the correlations and cluster analyses of the EOs of *P. aduncum* highlight dillapiole as a key compound. Several studies have demonstrated its insecticidal and antimicrobial activities, often identifying it as the major constituent [9,10,24,50]. This may explain the low incidence of herbivory observed in individuals under agroecological cultivation (personal observation but not quantified).

## 4. Materials and Methods

### 4.1. Origin and History of the Specimen

The cultivated *Piper aduncum* L. genotype originated from the propagation (stem cuttings) of a wild individual collected in the Tinguá Biological Reserve region (coordinates: 22°35′21.9″ S 43°16′44.5″ W, elevation: 28 m) in Xerém, Rio de Janeiro State, Brazil, October 2017, under collection authorizations granted by SISBIO (permit number 57296-1; authentication code 47749568) and CGEN (010771/2014-0). For seedling production, a healthy and vigorous wild matrix was selected, measuring 3 m in height. Three cuttings were taken from the secondary branches at a height of 1.40 m using pruning shears. Only one of the seedlings survived the quarantine period in the nursery. The surviving seedling was successfully transplanted into the Agroecological Cultivation Garden System at the Socio-Environmental Responsibility Center of the Rio de Janeiro Botanical Garden Research Institute (CRS/IPJBRJ) (coordinates: 22°58′01.2″ S 43°13′43.0″ W and elevation: 26m). Currently, the cultivated plant (genotype) is over five to six years old and approximately 8 m in height.

The cultivated plant was identified by Dr. Elsie Franklin Guimarães and Dr. George Azevedo Queiroz, both from the Rio de Janeiro Botanical Garden Research Institute (IPJBRJ). Herbarium samples were deposited in the RB/JBRJ Herbarium (RB01426180) as a voucher specimen. All plant description data were recorded, including height, leaf coloration, inflorescence/infructescence characteristics, and in situ climatic data [54].

Subsequent to the initial establishment of the focal plant, additional individuals have been obtained by vegetative propagation and are currently under cultivation to support future replicated analyses.

### 4.2. Harvesting and Extraction of the Essential Oils

To analyze the spatiotemporal influence on the production and chemical composition of essential oils (EOs) from *P. aduncum,* fresh leaves (50 g) were collected every 3 h over a 24 h period at 12:00 a.m., 3:00 a.m., 6:00 a.m., 9:00 a.m., 12:00 p.m., 3:00 p.m., 6:00 p.m., and 9:00 p.m. This procedure was conducted during both the dry (July 2023) and the rainy seasons (February 2024) [24]. A single *P. aduncum* individual was used in this study, and it was in the reproductive stage during both harvest periods.

Fresh *P. aduncum* leaves were subjected to hydrodistillation using a Clevenger-type apparatus, following a standard procedure previously described [11,24]. At the designated collection time, the leaves were weighed and immediately chopped manually with scissors. After 2 h of distillation [11], the EOs were separated from the aqueous phase, dried with anhydrous sodium sulfate (Na_2_SO_4_; Sigma-Aldrich, São Paulo, Brazil), filtered, and stored at −4 °C until analysis.

### 4.3. Qualitative and Quantitative Analysis of the Essential Oils

For chemical characterization of the EOs from fresh leaves of *P. aduncum*, the samples were solubilized in analytical-grade dichloromethane (Tedia, Rio de Janeiro, Brazil) until reaching a final concentration of approximately 1000 ppm. The solutions were then analyzed by Gas Chromatography–Mass Spectrometry (GC-MS) using an HP Agilent GC 6890—MS 5973(Agilent, Santa Clara, CA, USA) system to obtain mass spectra. To quantify the chemical compounds in the samples and to determine the linear retention index (LRI), GC coupled to a Flame Ionization Detector (GC-FID) was used (HP Agilent 6890 system). Both analyses were conducted at the Analytical Platform of Farmanguinhos, FIOCRUZ, Rio de Janeiro, Brazil.

The GC-MS and GC-FID analyses were carried out following parameters previously determined by Assunção et al. [11] for *P. aduncum* samples. The LRI of each compound in the analyzed sample was determined based on the retention time (Rt) of a homologous series of saturated aliphatic hydrocarbons (C_8_–C_28_, Sigma-Aldrich, São Paulo, Brazil) obtained by GC-FID under the same analytical conditions as the samples [55]. The compounds present in the EOs were identified by comparing their mass spectra with database records (WILEY 7*n*) and by comparing the calculated LRIs with those reported in the literature [56,57,58].

### 4.4. Equipment for Environmental Measures

During each harvest, meteorological microclimate variables were recorded for later analysis of potential correlations with the substances present in the EOs. The portable manual equipment used included: I—Digital Windmeter Anemometer Akrom KR825 (São Paulo, Brazil)—temperature (°C) and relative air humidity (%); II—Luxmeter Lux Meter (MT-30,Shenzhen, Guangdong, China))—light intensity (lux).

### 4.5. Statistical Treatment and Data Processing

Statistical analyses were performed to evaluate the relationships between the substances present in the EOs of *P. aduncum*. The data (triplicates) are presented as mean ± standard deviation. Analyses of variance (ANOVA) were performed using the Statistica software, version 12 (StatSoft Inc., Tulsa, OK, USA), to compare the means obtained in the results. Additionally, principal component analysis (PCA) and hierarchical cluster analysis (HCA) were conducted to assess variance with respect to collection time and chemical composition among the samples [24,59].

Pearson’s correlation, analyzed in Excel, was used to evaluate potential interrelationships between climatic variables (temperature, humidity, and light intensity) and selected EO compounds [24,55,59]. Correlation coefficients (positive or negative) were interpreted based on the following criteria: very weak (0.000 to 0.199), weak (0.200 to 0.399), moderate (0.400 to 0.699), strong (0.700 to 0.899), and very strong (0.900 to 1.000) [24,60].

## 5. Conclusions

This study shows that *Piper aduncum* essential oil production is temporally structured, with a clear diel pattern in essential oil yield and statistically supported time effects across seasons, indicating that harvest timing can be used as a practical strategy to improve productivity in cultivation systems. Although the magnitude of chemical richness differed between seasons, the essential oil profile remained strongly dominated by dillapiole throughout the circadian cycle, supporting the view that this species functions as a stable dillapiole-oriented production system even when environmental conditions shift. Correlation patterns among constituents further suggest regulated temporal tradeoffs among metabolic routes, consistent with a working model in which diel modulation can reshape the relative balance between dillapiole and other volatile fractions without displacing dillapiole as the central component of the essential oil.

Collectively, these results reinforce the relevance of chronobiology for applied natural products research, linking circadian dynamics to actionable agronomic decisions, while also highlighting the need to validate chronochemical patterns across additional individuals and genotypes to confirm the generality of these findings under field conditions.

## Figures and Tables

**Table 1:** Chemical composition and yields of essential oils from Piper aduncum leaves during the circadian cycle, conducted in the dry season in July 2023.

Compounds *	LRI_lit_	LRI_calc_	Relative Peak Area (%) ± SD							
			Dry Season (July)							
			12:00 a.m.	03:00 a.m.	06:00 a.m.	09:00 a.m.	12:00 p.m.	3:00 p.m.	6:00 p.m.	**9:00 p.m.**
α-pinene	932	942	-	0.09 ± 0.01	0.73 ± 0.05	-	0.11 ± 0.05	-	0.15 ± 0.04	0.52 ± 0.04
β-pinene	974	982	1.11 ± 0.01 ^e^	1.05 ± 0.02 ^ab^	1.85 ± 0.09 ^d^	1.50 ± 0.03 ^f^	1.62 ± 0.34 ^a^	- ^b^	1.26 ± 0.02 ^a^	1.45 ± 0.02 ^c^
α-phellandrene	1002	1006	-	0.07 ± 0.04	0.54 ± 0.02	-	0.56 ± 0.11	-	0.43 ± 0.00	0.06 ± 0.00
limonene	1024	1027	-	-	-	-	-	-	1.03 ± 0.22	-
*Z*-β-ocimene	1032	1032	1.13 ± 0.09 ^ab^	0.81 ± 0.12 ^a^	1.86 ± 0.35 ^d^	1.21 ± 0.03 ^ab^	1.17 ± 0.25 ^ab^	- ^c^	1.35 ± 0.05 ^ab^	1.26 ± 0.09 ^ab^
*E*-β-ocimene	1044	1043	2.49 ± 0.08 ^a^	2.58 ± 0.61 ^a^	4.67 ± 1.19 ^c^	2.97 ± 0.12 ^ab^	4.37 ± 0.06 ^bc^	- ^d^	2.96 ± 0.08 ^a^	3.25 ± 0.09 ^ab^
MW207	-	1367	-	-	-	-	-	-	0.07 ± 0.00	-
β-elemene	1389	1388	-	-	-	-	-	-	0.10 ± 0.01	-
*E*-caryophyllene	1417	1416	2.75 ± 0.01 ^f^	1.79 ± 0.01 ^b^	2.26 ± 0.02 ^d^	2.83 ± 0.01 ^g^	2.48 ± 0.04 ^a^	1.96 ± 0.01 ^c^	2.63 ± 0.02 ^e^	2.42 ± 0.05 ^a^
α-humulene	1452	1451	-	-	-	-	-	-	0.49 ± 0.01	0.42 ± 0.01
γ-muurolene	1478	1476	0.89 ± 0.01	0.55 ± 0.01	0.79 ± 0.07	0.96 ± 0.02	0.80 ± 0.03	0.74 ± 0.01	0.83 ± 0.01	0.61 ± 0.01
bicyclogermacrene	1500	1490	1.90 ± 0.01 ^c^	1.24 ± 0.13 ^b^	1.51 ± 0.12 ^a^	1.57 ± 0.05 ^a^	1.46 ± 0.09 ^ab^	1.41 ± 0.01 ^ab^	1.54 ± 0.02 ^a^	1.51 ± 0.18 ^a^
*n*-pentadecane	1500	1496	-	-	-	-	-	-	0.21 ± 0.00	0.19 ± 0.00
γ-cadinene	1513	1510	0.52 ± 0.03	0.71 ± 0.02	-	-	0.64 ± 0.03	0.79 ± 0.01	0.64 ± 0.01	0.52 ± 0.01
myristicin	1517	1513	-	-	-	-	-	-	0.40 ± 0.00	-
germacrene B	1559	1549	-	-	-	-	-	-	0.50 ± 0.00	-
*E*-nerolidol	1561	1555	-	-	-	-	-	-	0.26 ± 0.01	0.52 ± 0.01
MW222	-	1567	-	0.35 ± 0.04	-	-	-	-	0.14 ± 0.01	0.29 ± 0.00
MW206	-	1572	-	-	-	-	-	-	0.22 ± 0.01	-
globulol	1590	1584	1.06 ± 0.02 ^d^	1.22 ± 0.01 ^a^	1.24 ± 0.00 ^a^	1.18 ± 0.01 ^e^	1.30 ± 0.01 ^c^	1.23 ± 0.00 ^a^	1.28 ± 0.01 ^bc^	1.25 ± 0.02 ^e^
ledol	1602	1594	-	-	-	-	-	-	0.16 ± 0.00	-
**dillapiole**	**1620**	**1610**	**80.68 ± 0.21 ^a^**	**83.74 ± 0.14 ^c^**	**75.78 ± 0.60 ^a^**	**80.16 ± 0.11 ^a^**	**80.35 ± 0.76 ^a^**	**88.27 ± 0.03 ^d^**	**79.75 ± 0.39 ^a^**	**80.87 ± 0.36 ^a^**
*epi*-α-muurolol	1640	1643	-	-	-	-	-	-	0.19 ± 0.00	0.20 ± 0.01
**Non-oxygenated monoterpenes**			**4.72**	**4.60**	**9.65**	**5.68**	**6.47**	**-**	**7.18**	**6.55**
**Oxygenated monoterpenes**			**-**	**-**	**-**	**-**	**-**	**-**	**-**	**-**
**Non-oxygenated sesquiterpenes**			**6.05**	**4.28**	**4.56**	**5.36**	**5.38**	**4.91**	**6.23**	**5.15**
**Oxygenated sesquiterpenes**			**1.06**	**1.22**	**1.84**	**1.18**	**1.30**	**1.23**	**1.90**	**1.98**
**Arylpropanoids**			**80.68**	**83.74**	**75.78**	**80.16**	**80.35**	**88.27**	**80.15**	**80.87**
**Other compounds**			**-**	**0.35**	**-**	**-**	**-**	**-**	**0.63**	**0.81**
**Number of identified compounds**			**09**	**11**	**11**	**08**	**11**	**06**	**20**	**15**
**Total identified (%)**			**92.52**	**94.19**	**91.84**	**92.39**	**94.84**	**94.40**	**96.59**	**95.37**
**Essential oil Yield (%)**			**0.71 ± 0.04 ^a^**	**0.24 ± 0.01 ^b^**	**0.47 ± 0.01 ^c^**	**0.54 ± 0.02 ^d^**	**0.49 ± 0.01 ^e^**	**0.73 ± 0.01 ^a^**	**0.59 ± 0.02 ^f^**	**0.69 ± 0.01 ^g^**

Legend: LIR_calc_ = Calculated Linear Retention Index (HP-5MS column); LRI_lit_ = Literature Linear Retention index (Adams, 2017 [57]); Main constituents in bold. * = Quantities are averaged out of three replicates. ± = Standard deviation (SD). All compounds were identified by MS and LRI in accordance with experimental methods; MW = molecular weight; Means followed by the same letter in a row are not significantly different (Tukey test, *p* < 0.05).

**Table 2:** Chemical composition and yields of essential oils from *Piper aduncum* leaves during the circadian cycle, conducted in the rainy season in February 2024.

Compounds *	LRI_lit_	LRI_calc_	Relative Peak Area (%) ± SD							
			Rainy Season (February)							
			12:00 a.m.	03:00 a.m.	06:00 a.m.	09:00 a.m.	12:00 p.m.	3:00 p.m.	6:00 p.m.	9:00 p.m.
β-pinene	974	983	0.78 ± 0.01	0.63 ± 0.02	0.83 ± 0.02	-	-	-	-	0.90 ± 0.00
*Z*-β-ocimene	1032	1036	0.94 ± 0.01 ^a^	0.87 ± 0.02 ^b^	0.64 ± 0.00 ^c^	- ^d^	- ^d^	- ^d^	2.34 ± 0.04 ^e^	0.86 ± 0.02 ^b^
*E*-β-ocimene	1044	1046	2.24 ± 0.04 ^a^	2.78 ± 0.04 ^b^	1.16 ± 0.01 ^c^	2.60 ± 0.02 ^d^	1.45 ± 0.02 ^e^	- ^f^	0.89 ± 0.03 ^g^	0.57 ± 0.01 ^h^
MW429	-	1322	0.54 ± 0.01	0.51 ± 0.00	0.65 ± 0.03	-	1.03 ± 0.01	-	0.51 ± 0.07	0.23 ± 0.00
*E*-caryophyllene	1417	1420	3.00 ± 0.02 ^a^	2.62 ± 0.02 ^b^	4.21 ± 0.01 ^c^	3.30 ± 0.07 ^d^	3.72 ± 0.01 ^e^	2.87 ± 0.02 ^f^	3.07 ± 0.06 ^b^	2.57 ± 0.00 ^b^
MW141	-	1448	-	-	-	-	-	-	-	0.15 ± 0.00
*cis*-cadina-1(6).4-diene	1476	1461	0.58 ± 0.01	0.29 ± 0.04	0.41 ± 0.01	0.32 ± 0.05	0.72 ± 0.01	-	0.28 ± 0.07	0.30 ± 0.01
bicyclogermacrene	1500	1492	2.49 ± 0.03 ^a^	2.10 ± 0.03 ^d^	2.52 ± 0.05 ^ab^	2.68 ± 0.01 ^f^	2.24 ± 0.02 ^e^	1.63 ± 0.01 ^c^	2.45 ± 0.05 ^a^	2.57 ± 0.01 ^b^
MW503	-	1502	0.85 ± 0.02	0.19 ± 0.01	0.28 ± 0.00	0.85 ± 0.17	0.29 ± 0.01	-	0.20 ± 0.01	0.17 ± 0.02
γ-cadinene	1513	1511	-	-	0.54 ± 0.00	-	-	-	-	0.33 ± 0.03
δ-cadinene	1522	1520	- ^a^	- ^a^	- ^a^	- ^a^	1.00 ± 0.02 ^b^	4.58 ± 0.07 ^c^	- ^a^	0.34 ± 0.01 ^d^
aromadendrene	1425	1439	-	-	-	-	-	0.04 ± 0.02	-	-
MW204	-	1586	0.82 ± 0.01	0.48 ± 0.01	0.76 ± 0.01	0.46 ± 0.02	0.77 ± 0.01	-	0.53 ± 0.01	0.58 ± 0.00
**dillapiole**	**1620**	**1618**	**78.03 ± 0.50 ^ab^**	**82.77 ± 0.29 ^f^**	**75.90 ± 0.16 ^e^**	**78.69 ± 0.19 ^cd^**	**77.80 ± 0.03 ^ab^**	**90.86 ± 0.09 ^g^**	**77.71 ± 0.13 ^a^**	**79.50 ± 0.64 ^d^**
MW401	-	1673	-	0.60 ± 0.02	0.74 ± 0.00	0.39 ± 0.00	0.77 ± 0.01	-	1.05 ± 0.01	1.07 ± 0.01
**Non-oxygenated monoterpenes**			**3.96**	**4.28**	**2.63**	**2.60**	**1.45**	**-**	**3.74**	**2.33**
**Oxygenated monoterpenes**			**-**	**-**	**-**	**-**	**-**	**-**	**-**	**-**
**Non-oxygenated sesquiterpenes**			**6.07**	**5.01**	**5.16**	**6.30**	**7.68**	**9.12**	**5.80**	**6.11**
**Oxygenated sesquiterpenes**			**-**	**-**	**-**	**-**	**-**	**-**	**-**	**-**
**Arylpropanoids**			**78.03**	**82.77**	**75.90**	**78.69**	**77.80**	**90.86**	**77.71**	**79.50**
**Other compounds**			**2.21**	**1.78**	**2.43**	**1.70**	**2.86**	**-**	**2.29**	**2.20**
**Number of identified compounds**			**07**	**07**	**08**	**05**	**06**	**05**	**06**	**09**
**Total identified (%)**			**90.25**	**93.85**	**88.64**	**89.29**	**89.78**	**99.98**	**89.02**	**90.14**
**Essential oil Yield (%)**			**0.33 ± 0.01 ^a^**	**0.23 ± 0.03 ^b^**	**0.38 ± 0.01 ^c^**	**0.33 ± 0.02 ^a^**	**0.61 ± 0.03 ^d^**	**0.32 ± 0.04 ^a^**	**0.29 ± 0.02 ^e^**	**0.24 ± 0.01 ^b^**

Legend: LIR_calc_ = Calculated Linear Retention Index (HP-5MS column); LRI_lit_ = Literature Linear Retention index (Adams, 2017 [57]); Main constituents in bold. * = Quantities are averaged out of three replicates. ± = Standard deviation (SD). All compounds were identified by MS and LRI in accordance with experimental methods; MW = molecular weight; Means followed by the same letter in a row are not significantly different (Tukey test, *p* < 0.05).

**Table 3:** Pearson correlation between compounds, environmental variables, and essential oil yields of *Piper aduncum* in the circadian cycle of July 2023.

**Dry season**	**Correlations: Compounds vs. Compounds**									
		β-pinene	limonene	*Z*-β-ocimene	*E*-β-ocimene	*E*-caryophyllene	bicyclogermacrene	globulol	ledol	dillapiole
	β-pinene	1.000								
	limonene	0.022	1.000							
	*Z*-β-ocimene	0.942	0.191	1.000						
	*E*-β-ocimene	0.973	0.014	0.908	1.000					
	*E*-caryophyllene	0.469	0.263	0.495	0.341	1.000				
	bicyclogermacrene	0.167	0.049	0.305	0.080	0.781	1.000			
	globulol	−0.384	0.246	−0.545	−0.404	0.040	−0.211	1.000		
	ledol	0.452	−0.014	0.610	0.508	−0.110	−0.010	−0.925	1.000	
	dillapiole	−0.932	−0.163	−0.992	−0.904	−0.533	−0.356	0.586	−0.638	1.000
	**Correlations: Compounds vs. Environmental variables**									
		β-pinene	limonene	*Z*-β-ocimene	*E*-β-ocimene	*E*-caryophyllene	bicyclogermacrene	globulol	ledol	dillapiole
	Temperature	−0.402	0.307	−0.535	−0.408	0.102	−0.233	0.732	−0.630	0.504
	Humidity	0.035	−0.477	−0.207	−0.031	0.000	−0.107	0.558	−0.678	0.268
	Lux	−0.384	−0.249	−0.532	−0.399	0.045	−0.164	0.296	−0.293	0.455

Legend: Colors indicate correlation levels, with darker shades representing stronger correlations. Blue corresponds to negative correlations, while red indicates positive correlations. Correlation ranges are defined as follows: very weak (0 to 0.199), weak (0.200 to 0.399), moderate (0.400 to 0.699), strong (0.700 to 0.899), and very strong (0.900 to 1.000).

**Table 4:** Pearson correlation between compounds, environmental variables, and essential oil yields of *Piper aduncum* in the circadian cycle of February 2024.

**Rainy season**	**Correlations: Compounds vs. Compounds**						
		*Z*-β-ocimene	*E*-β-ocimene	*E*-caryophyllene	bicyclogermacrene	δ-cadinene	dillapiole
	Z-β-ocimene	1.000					
	*E*-β-ocimene	−0.076	1.000				
	*E*-caryophyllene	−0.236	0.000	1.000			
	bicyclogermacrene	0.286	0.381	0.261	1.000		
	δ-cadinene	−0.447 *	−0.630 *	−0.165	−0.858 *	1.000	
	dillapiole	−0.336	−0.368	−0.514	−0.885 *	0.877 *	1.000
	**Correlations: Compounds vs. Environmental variables**						
		*Z*-β-ocimeno	*E*-β-ocimeno	*E*-caryophyllene	bicyclogermacrene	δ-cadineno	dillapiole
	Temperature	−0.603 *	−0.014	0.352	−0.315	0.346	0.083
	Humidity	0.488 *	0.348	−0.161	0.498 *	−0.585 *	−0.302
	Lux	−0.523 *	0.036 *	0.883 *	0.353	−0.186	−0.477 *
	**Correlations: Chemical classes vs. Environmental variables**						
		Non-oxygenated monoterpenes	Non-oxygenated sesquiterpenes		Arilpropanoids		Other compounds
	Temperature	−0.531 *	0.501 *		0.083		0.095
	Humidity	0.725 *	−0.620 *		−0.302		0.098
	Lux	−0.529 *	0.403 *		0.125		−0.154
		Temperature	Humidity	Lux			
	Yield	0.892 *	−0.764 *	0.581 *			

Legend: Colors indicate correlation levels, with darker shades representing stronger correlations. Blue corresponds to negative correlations, while red indicates positive correlations. Correlation ranges are defined as follows: very weak (0 to 0.199), weak (0.200 to 0.399), moderate (0.400 to 0.699), strong (0.700 to 0.899), and very strong (0.900 to 1.000). * = Significant at *p* < 0.05.

**Table 5:** Pearson correlation between the identified compounds in the essential oils of *Piper aduncum* leaves collected in July 2023 (dry season) and February 2024 (rainy season).

	**Correlations: Compounds vs. Compounds**						
		**Rainy season**					
		*Z*-β-ocimene	*E*-β-ocimene	*E*-caryophyllene	bicyclogermacrene	δ-cadinene	dillapiole
**Dry season**	β-pinene	0.126	0.348	0.566 *	0.835 *	−0.825 *	−0.937 *
	limonene	0.845 *	−0.231	−0.072	0.137	−0.188	−0.208
	Z-β-ocimene	0.341	0.231	0.579 *	0.860 *	−0.829 *	−0.954 *
	*E*-β-ocimene	0.109	0.270	0.640 *	0.699 *	−0.744 *	−0.899 *
	*E*-oaryophyllene	0.159	0.191	0.227	0.746 *	−0.455 *	−0.640 *
	bicyclogermacrene	0.157	0.112	0.123	0.506 *	−0.267	−0.429 *
	globulol	0.056	−0.037	−0.671 *	−0.281	0.238	0.364
	ledol	0.076	−0.153	0.749 *	0.240	−0.214	−0.393
	dillapiole	−0.276	−0.231	−0.664 *	−0.843 *	0.787 *	0.953 *

Legend: Colors indicate correlation levels, with darker shades representing stronger correlations. Blue corresponds to negative correlations, while red indicates positive correlations. Correlation ranges are defined as follows: very weak (0 to 0.199), weak (0.200 to 0.399), moderate (0.400 to 0.699), strong (0.700 to 0.899), and very strong (0.900 to 1.000). * = Significant at *p* < 0.05.

## Data Availability

The raw data supporting the findings of this study can be obtained from the corresponding author upon request.

## Supplementary Materials

The following supporting information can be downloaded at: https://www.mdpi.com/article/10.3390/plants15060976/s1, Figure S1: Mass spectrum of the single major compound present in the essential oils from the circadian cyrcle conducted during the dry and rainy seasons; Figure S2: Principal component analysis (PCA) of the essential oils from Piper aduncum leaves throughout the circadian cyrcle, in the dry (S) and rainy (C) seasons over a 24-hours period.
