# Supplementary material for: Temporal Variation in the Essential Oil Production of Piper aduncum L.: Influence of Circadian Rhythms and Insights into Dillapiole Production Dynamics
Source: Plants (Basel). 2026 Mar 21;15(6):976. doi: 10.3390/plants15060976 (PMC13029857; doi:10.3390/plants15060976)
Supplement: Supplementary file 1 [file plants-15-00976-s001.zip › plants-4167078-supplementary.pdf]

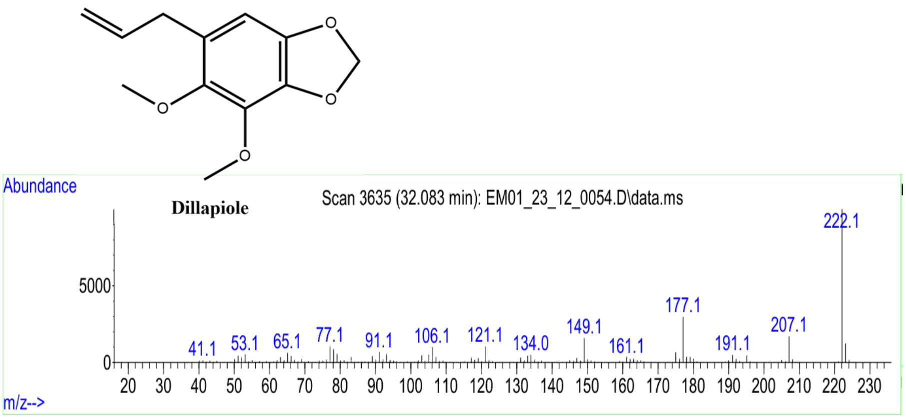

Supplementary Material Figure S1: Mass spectrum of the single major compound present in the essential oils from the circadian cycle conducted during the dry and rainy seasons.

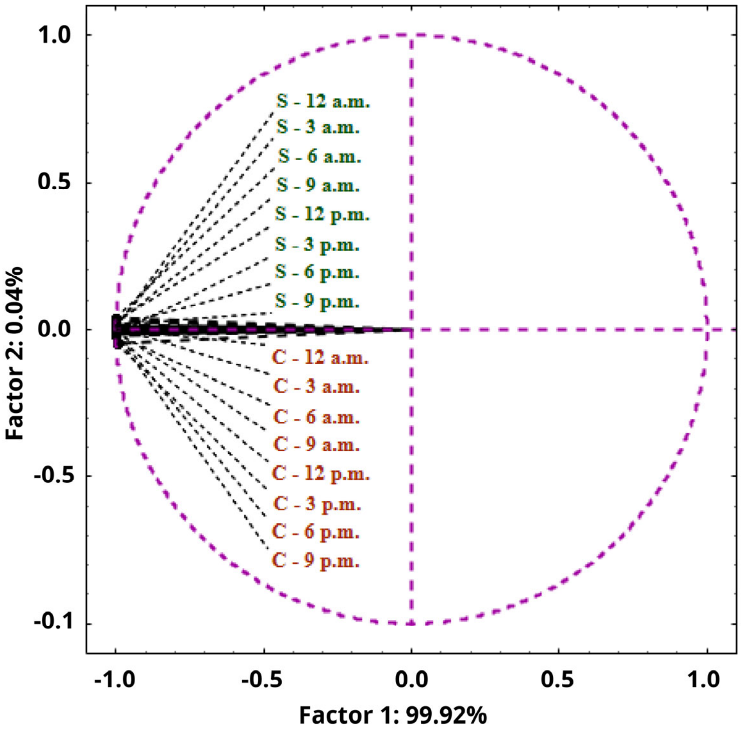

Supplementary Material Figure S2: Principal Component Analysis (PCA) of the essential oils from *Piper aduncum* leaves throughout the circadian cycle, in the dry (S) and rainy (C) seasons over a 24-hour period.
